# Proposed New Constitution and By-Laws

**Published:** 1905-03

**Authors:** 


### PROPOSED NEW CONSTITUTION AND BY-LAWS.

THE following Constitution and By-Laws has been suggested as a uniform style to be adopted by the different State associations. It will come before the Association for adoption or rejection at its next meeting in Atlanta in 1905. It is here published in full that the members may consider it carefully and be in a position to vote intelligently upon it.

### CONSTITUTION.

#### ARTICLE I.—NAME OF THE ASSOCIATION.

The name and title of this organization shall be The Medical Association of Georgia.

#### ARTICLE II.—PURPOSES OF THE ASSOCIATION.

The purposes of this Association shall be to federate and bring into one compact organization the entire medical profession of the State of Georgia, and to unite with similar societies of other States to form the American Medical Association; to extend medical knowledge and advance medical science; to elevate the standard of medical education, and to secure the enactment and enforcement of just medical laws; to promote friendly intercourse among physicians; to guard and foster the material interests of its members and to protect them against imposition ; and to enlighten and direct public opinion in regard to the great problems of State medicine, so that the profession shall become more capable and honorable within itself, and more useful to the public, in the prevention and cure of disease, and in prolonging and adding comfort to life.

ARTICLE III.—COMPONENT SOCIETIES.

Component Societies shall consist of those county medical societies which hold charters from this Association.

ARTICLE IV.—COMPOSITION OF THE ASSOCIATION.

SECTION 1. This Association shall consist of Members, Delegates and Guests.

SEC. 2. MEMBERS. The members of this Association shall be the members of the component county medical societies to which only white physicians shall be eligible.

SEC. 3. DELEGATES. Delegates shall be those members who are elected in accordance with this Constitution and By-Laws to represent their respective component societies in the House of Delegates of this Association.

SEC. 4. GUESTS. Any distinguished physician not a resident of this State who is a member of his own State Association may become a guest during any Annual Session on invitation of the officers of this Association, and shall be accorded the privilege of participating in all of the scientific work for that session.

ARTICLE V.—HOUSE OF DELEGATES.

The House of Delegates shall be the legislative and business body of the Association, and shall consist of (1) Delegates elected by the component county societies, (2) the Councilors, and (3) *ex officio*, the President and Secretary of this Association.

ARTICLE VI.—COUNCIL.

The Council shall consist of the Councilors, and the President and Secretary, *ex officio*. Besides its duties mentioned in the By-Laws, it shall constitute the Finance Committee of the House of Delegates. Five Councilors shall constitute a quorum.

### ARTICLE VII.—SECTIONS AND DISTRICT SOCIETIES.

The House of Delegates may provide for a division of the scientific work of the Association into appropriate sections, and for the organization of such Councilor District Societies as will promote the best interests of the profession, such societies to be composed exclusively of members of component societies.

### ARTICLE VIII.—SESSIONS AND MEETINGS.

SECTION 1. The Association shall hold an annual session, during which there shall be held daily General Meetings, which shall be open to all registered members and guests.

SEC. 2. The time and place for holding each annual session shall be fixed by the House of Delegates.

### ARTICLE IX.—OFFICERS.

SECTION 1. The officers of this Association shall be a President, three Vice-Presidents, a Secretary and Treasurer, and twelve Councilors.

SEC. 2. The officers, except the Secretary-Treasurer and Councilors, shall be elected annually. The President shall appoint the first Councilors to serve for one year, or until their successors are elected. The terms of the elected Councilors shall be for three years, those first elected serving one, two and three years, as may be arranged. The Secretary and Treasurer shall be elected for a term of five years. All these officers shall serve until their successors are elected and installed.

SEC. 3. The officers of this Association except the President, shall be elected by the House of Delegates on the morning of the last day of the annual session, but no delegate shall be eligible to any office named in the preceding section, except that of Councilor, and no person shall be elected to any such office, who is not in attendance upon that annual session, and who has not been a member of the Association for the past two years. The President shall be elected in the body of the Association by ballot.

### ARTICLE X.—RECIPROCITY OF MEMBERSHIP WITH OTHER STATE SOCIETIES.

In order to broaden professional fellowship this Association is ready to arrange with other State Medical Associations for an interchange of certificates of membership, so that members moving from one State to another may avoid the formality of re-election.

ARTICLE XI.—FUNDS AND EXPENSES.

Funds shall be raised by an equal per capita assessment on each component society. The amount of the assessment shall be fixed by the House of Delegates, but shall not exceed the sum of $3.00 per capita per annum, except on a four-fifths vote of the delegates present. Funds may also be raised by voluntary contributions, from the Associations, and in any other manner approved by the House of Delegates. Funds may be appropriated by the House of Delegates to defray the expenses of the Association, for publications, and for such other purposes as will promote the welfare of the profession. All resolutions appropriating funds must be referred to the Finance Committee before action is taken thereon.

ARTICLE XII.—REFERENDUM.

SECTION 1. A general meeting of the Association may, by a two-thirds vote of the members present, order a general referendum on any qusestion pending before the House of Delegates, and when so ordered the House of Delegates shall submit such question to the members of the Association, who may vote by mail or in person, and, if the members voting shall comprise a majority of all the members of the Association, a majority of such vote shall determine the question and be binding on the House of Delegates.

SEC. 2. The House of Delegates may, by a two-thirds vote of its own members, submit any question before it to a general referendum, as provided in the preceding section, and the result shall be binding on the House of Delegates.

ARTICLE XIII.—THE SEAL.

The Association shall have a common Seal, with power to break, change or renew the same at pleasure.

ARTICLE XIV.—AMENDMENTS.

The House of Delegates may amend any article of this Constitution by a two-thirds vote of the delegates present at any annual session, provided that such amendment shall have been presented in open meeting at the previous annual session, and that it shall have been published twice during the year in the bulletin or journal of this Association, or sent officially to each component society at least two months before the meeting at which final action is to be taken.

### BY-LAWS.

### CHAPTER I.—MEMBERSHIP.

SECTION 1. The name of a physician on the properly certified roster of members of a component society, which has paid its annual assessment, shall be *prima facie* evidence of membership in this Association.

SEC. 2. Any person who is under sentence of suspension or expulsion from a component society, or whose name has been dropped from its roll of members, shall not be entitled to any of the rights or benefits of this Association, nor shall he be permitted to take part in any of its proceedings until he has been relieved of such disability.

SEC. 3. Each member in attendance at the annual session shall enter his name on the registration book, indicating the component society of which he is a member. When his right to membership has been verified, by reference to the roster of his society, he shall receive a badge, which shall be evidence of his right to all the privileges of membership at that session. No member shall take part in any of the proceedings of an annual session until he has complied with the provisions of this section.

### CHAPTER II.—ANNUAL AND SPECIAL SESSIONS OF THE ASSOCIATION.

SECTION 1. The Association shall hold an Annual Session at such time and place as has been fixed at the preceding Annual Session by the House of Delegates.

SEC. 2. Special meetings of either the Association or of the House of Delegates shall be called by the President on petition of twenty delegates or fifty members.

### CHAPTER III.—GENERAL MEETINGS.

SECTION 1. All registered members may attend and participate in the proceedings and discussions of the General Meetings and of the Sections. The General Meetings shall be presided over by the President or by one of the Vice-Presidents, and before them shall be delivered the address of the President and the orations.

SEC. 2. The General Meeting may recommend to the House of Delegates the appointment of committees or commissions for scientific investigation of special interest and importance to the profession and public.

### CHAPTER IV.—HOUSE OF DELEGATES.

SECTION 1. The House of Delegates shall meet at 2 p.m. on the day before that fixed as the first day of the annual session. It may adjourn from time to time as may be necessary to complete its business; provided, that its hours shall conflict as little as possible with the General Meetings. The order of business shall be arranged as a separate section of the program.

SEC. 2. Each component county society shall be entitled to send to the House of Delegates each year one delegate for every fifty members, and one for each fraction thereof, but each component society which has made its annual report and paid its assessment as provided in this Constitution and By-Laws, shall be entitled to one delegate.

SEC. 3. Twenty-five delegates shall constitute a quorum.

SEC. 4. It shall, through its officers, Council and otherwise, give diligent attention to and foster the scientific work and spirit of the Association, and shall constantly study and strive to make each annual session a stepping-stone to future ones of higher interest.

SEC. 5. It shall consider and advise as to the material interests of the profession, and of the public, in those important matters wherein it is dependent upon the profession, and shall use its influence to secure and enforce all proper medical and public-health legislation, and to diffuse popular information in relation thereto.

SEC. 6. It shall make careful inquiry into the condition of the profession of each county in the State, and shall have authority to adopt such methods as may be deemed most efficient for building up and increasing the interest in such county societies as already exist, and for organizing the profession in counties where societies do not exist. It shall especially and systematically endeavor to promote friendly intercourse among physicians of the same locality, and shall continue these efforts until every physician in every county of the State who can be made reputable has been brought under medical society influence.

SEC. 7. It shall encourage postgraduate and research work, as well as home study, and shall endeavor to have the results utilized and intelligently discussed in the county societies.

SEC. 8. It shall elect representatives to the House of Delegates of the American Medical Association in accordance with the Constitution and By-Laws of that body.

SEC. 9. It shall divide the State into Councilor Districts, specifying what counties each district shall include, and, when the best interest of the Association and profession will be promoted thereby, organize in each a district medical society, and all members of component county societies, and no others shall be members in such district societies. When so organized, from the presidents of such district societies shall be chosen the vice-presidents of this Association, and the presidents of the county societies of the district shall be the vice-presidents of such district societies.

SEC. 10. It shall have authority to appoint committees for special purposes from among members of the Association who are not members of the House of Delegates. Such committees shall report to the House of Delegates, and may be present and participate in the debate on their reports.

SEC. 11. It shall approve all memorials and resolutions issued in the name of the Association before the same shall become effective.

(Concluded in April issue.)

